# The genome sequence of a parasitoid wasp, *Ichneumon xanthorius *Forster, 1771

**DOI:** 10.12688/wellcomeopenres.17683.1

**Published:** 2022-02-10

**Authors:** University of Oxford and Wytham Woods Genome Acquisition Lab, Gavin Broad, Darwin Tree of Life Barcoding collective, Wellcome Sanger Institute Tree of Life programme, Wellcome Sanger Institute Scientific Operations: DNA Pipelines, Tree of Life Core Informatics collective, Darwin Tree of Life Consortium

**Affiliations:** 1 Department of Life Sciences, Natural History Museum, London, UK

**Keywords:** Ichneumon xanthorius, genome sequence, chromosomal, Hymenoptera

## Abstract

We present a genome assembly from an individual female *Ichneumon xanthorius *(Arthropoda; Insecta; Hymenoptera; Ichneumonidae). The genome sequence is 315 megabases in span. The majority of the assembly (82.64%) is scaffolded into 12 chromosomal pseudomolecules. Gene annotation of this assembly on Ensembl has identified 10,622 protein coding genes.

## Species taxonomy

Eukaryota; Metazoa; Ecdysozoa; Arthropoda; Hexapoda; Insecta; Pterygota; Neoptera; Endopterygota; Hymenoptera; Apocrita; Parasitoida; Ichneumonoidea; Ichneumonidae; Ichneumoninae; Ichneumon; *Ichneumon xanthorius* Forster, 1771 (NCBI:txid2795680).

## Background

*Ichneumon xanthorius* is an idiobiont endoparasitoid of Lepidoptera, ovipositing in the host pupa, arresting its development, consuming it entirely and pupating within the host pupal shell. It has been reared from pupae of several species of *Noctua* Linnaeus and *Xestia* Hübner (Lepidoptera: Noctuidae) in experiments where females were presented with potential hosts ( Hinz & Horstmann, 2007); although the natural host range is poorly known, it will be limited to noctuid moths with larvae which feed low in the vegetation and pupate in the spring. This is a univoltine species, with females active post-hibernation mainly in April and May and then the next generation from July to September, sometimes into October, before spending the winter in concealed locations, such as under bark. Males are seen from mid-June to late August, mostly in July (data from iRecord; requires registration) and do not over-winter. The species is widely distributed across Europe, North Africa, the Middle East and into Central Asia. In Britain, *I. xanthorius* seems to be most frequent in southern England but has also been widely recorded in Scotland, Wales and Ireland ( Broad, 2016; O’Connor *et al.*, 2007; data from iRecord). Found mainly in open areas, including gardens, and often seen on flowers, especially umbels.

In common with many species of the subfamily Ichneumoninae, *Ichneumon xanthorius* is strongly sexually dimorphic, although both females and males have distinctive colour patterns which mean the species is unlikely to be confused with any other in Northern Europe. Females have a yellow- and black-striped metasoma (abdomen minus the first segment, which is fused with the thorax in apocritan Hymenoptera), males have the metasoma extensively yellow anteriorly with a characteristic large yellow spot on the hind coxa too.

To our knowledge, this is the first chromosomal genome produced for an ichneumonid wasp. The Ichneumonidae, or Darwin Wasps, comprise one of the great radiations of metazoan life, with over 25,000 described (and many more undescribed) species attacking a huge variety of (mostly) holometabolous insects ( Broad *et al.*, 2018; Klopfstein *et al.*, 2019). Genomic data will help us uncover some of the adaptations that have enabled this success.

##  Genome sequence report

**Figure 1.**
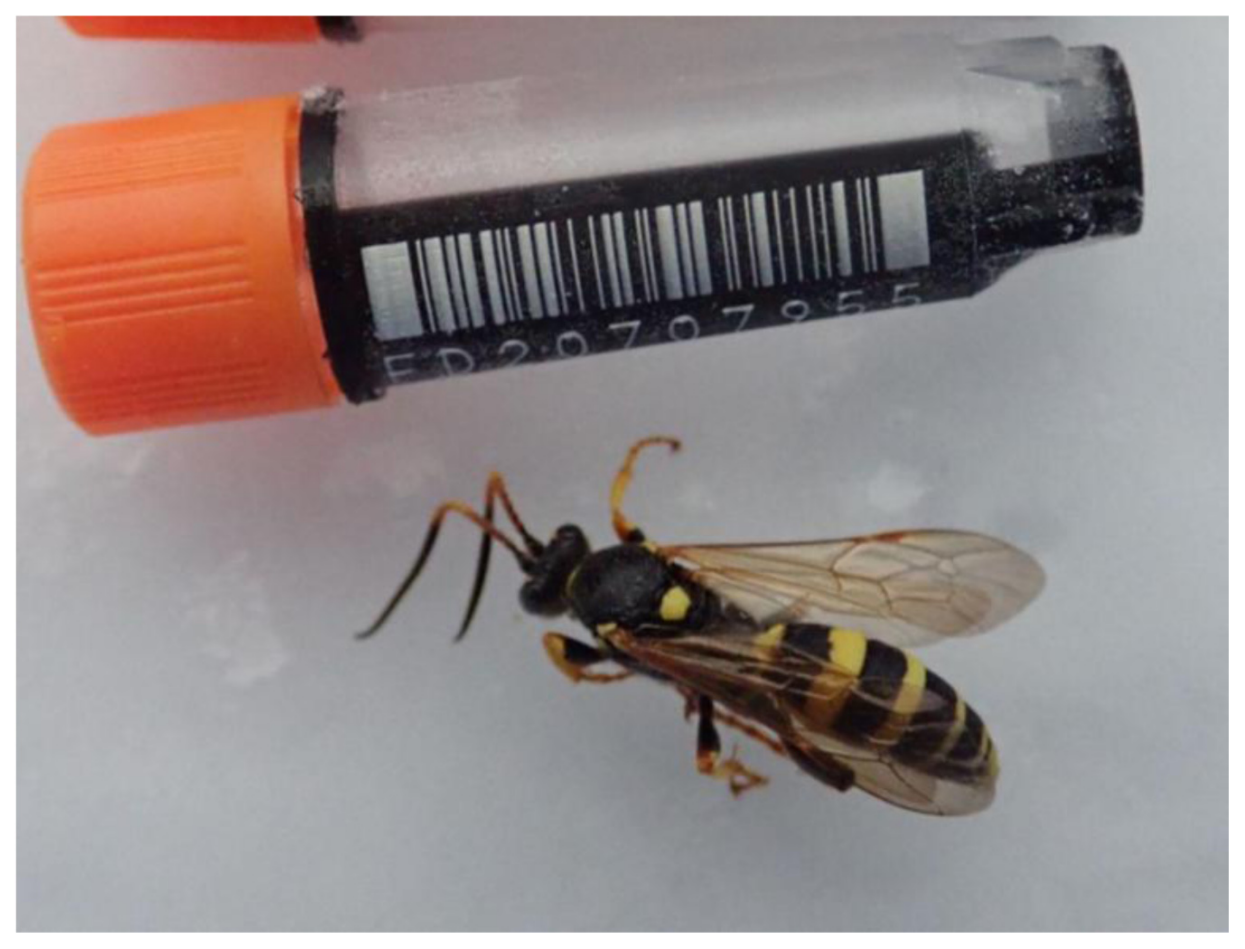
Image of the iyIchXant1 specimen taken during preservation and processing.

The genome was sequenced from a single female *I. xanthorius* ( Figure 1) collected from Wytham Woods, Oxfordshire (biological vice-county: Berkshire), UK (latitude 51.770, longitude -1.331). A total of 55-fold coverage in Pacific Biosciences single-molecule long reads and 62-fold coverage in 10X Genomics read clouds were generated. Primary assembly contigs were scaffolded with chromosome conformation Hi-C data. Manual assembly curation corrected 85 missing/misjoins and removed 1 haplotypic duplication, reducing the assembly size by 0.05% and scaffold number by 32.13%, and increasing the scaffold N50 by 57.52%.

**Figure 2.**
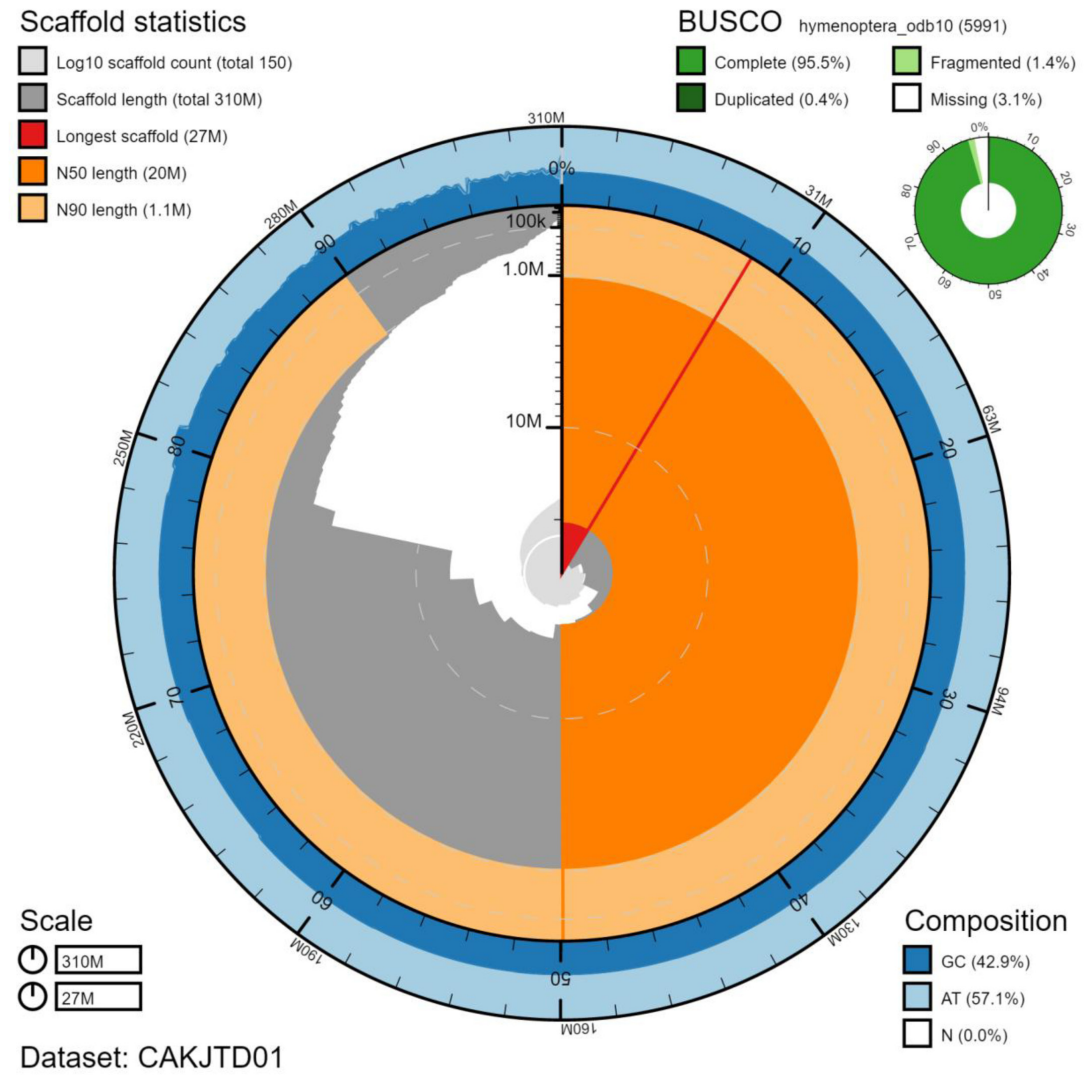
Genome assembly of *Ichneumon xanthorius*, iyIchXant1.1: metrics. The BlobToolKit Snailplot shows N50 metrics and BUSCO gene completeness.The main plot is divided into 1,000 size-ordered bins around the circumference with each bin representing 0.1% of the 314,990,913 bp assembly. The distribution of scaffold lengths is shown in dark grey with the plot radius scaled to the longest scaffold present in the assembly (27,402,130 bp, shown in red). Orange and pale-orange arcs show the N50 and N90 scaffold lengths (20,145,675 and 1,060,478 bp), respectively. The pale grey spiral shows the cumulative scaffold count on a log scale with white scale lines showing successive orders of magnitude. The blue and pale-blue area around the outside of the plot shows the distribution of GC, AT and N percentages in the same bins as the inner plot. A summary of complete, fragmented, duplicated and missing BUSCO genes in the hymenoptera_odb10 set is shown in the top right. An interactive version of this figure is available at https://blobtoolkit.genomehubs.org/view/iyIchXant1.1/dataset/CAKJTD01/snail.

The final assembly has a total length of 315 Mb in 150 sequence scaffolds with a scaffold N50 of 20.1 Mb ( Table 1). Of the assembly sequence, 82.64% was assigned to 12 chromosomal-level scaffolds (numbered by sequence length) ( Figure 2– Figure 5; Table 2). The orientation of chromosome 10, region 1.08–1.74 Mb cannot be determined from available data. The assembly has a BUSCO v5.1.2 ( Manni *et al.*, 2021) completeness of 95.5% (single 95.2%, duplicated 0.4%) using the hymenoptera_odb10 reference set (n=5991). While not fully phased, the assembly deposited is of one haplotype. Contigs corresponding to the second haplotype have also been deposited.

**Table 1.** Genome data for *Ichneumon xanthorius*, iyIchXant1.1.

*Project accession data*	
Assembly identifier	iyIchXant1.1
Species	*Ichneumon xanthorius*
Specimen	iyIchXant1
NCBI taxonomy ID	NCBI:txid2795680
BioProject	PRJEB46331
BioSample ID	SAMEA7746465
Isolate information	Female, head/thorax
*Raw data accessions*	
PacificBiosciences SEQUEL II	ERR6808013
10X Genomics Illumina	ERR6688586-ERR6688589
Hi-C Illumina	ERR6688585
*Genome assembly*	
Assembly accession	GCA_910589235.1
*Accession of alternate haplotype*	GCA_910589515.1
Span (Mb)	230
Number of contigs	214
Contig N50 length (Mb)	3
Number of scaffolds	106
Scaffold N50 length (Mb)	10
Longest scaffold (Mb)	25
BUSCO * genome score	C:95.5%[S:95.2%,D:0.4%],F:1.4%,M:3.1%,n:5991
*Genome annotation*	
Number of protein-coding genes	10,622
Average length of coding sequence (bp)	1,454.23
Average number of exons per transcript	6.14
Average exon size (bp)	279.63
Average intron size (bp)	1,109.68

*BUSCO scores based on the hymenoptera_odb10 BUSCO set using v5.1.2. C= complete [S= single copy, D=duplicated], F=fragmented, M=missing, n=number of orthologues in comparison. A full set of BUSCO scores is available at https://blobtoolkit.genomehubs.org/view/iyIchXant1.1/dataset/CAKJTD01/busco.

**Figure 3.**
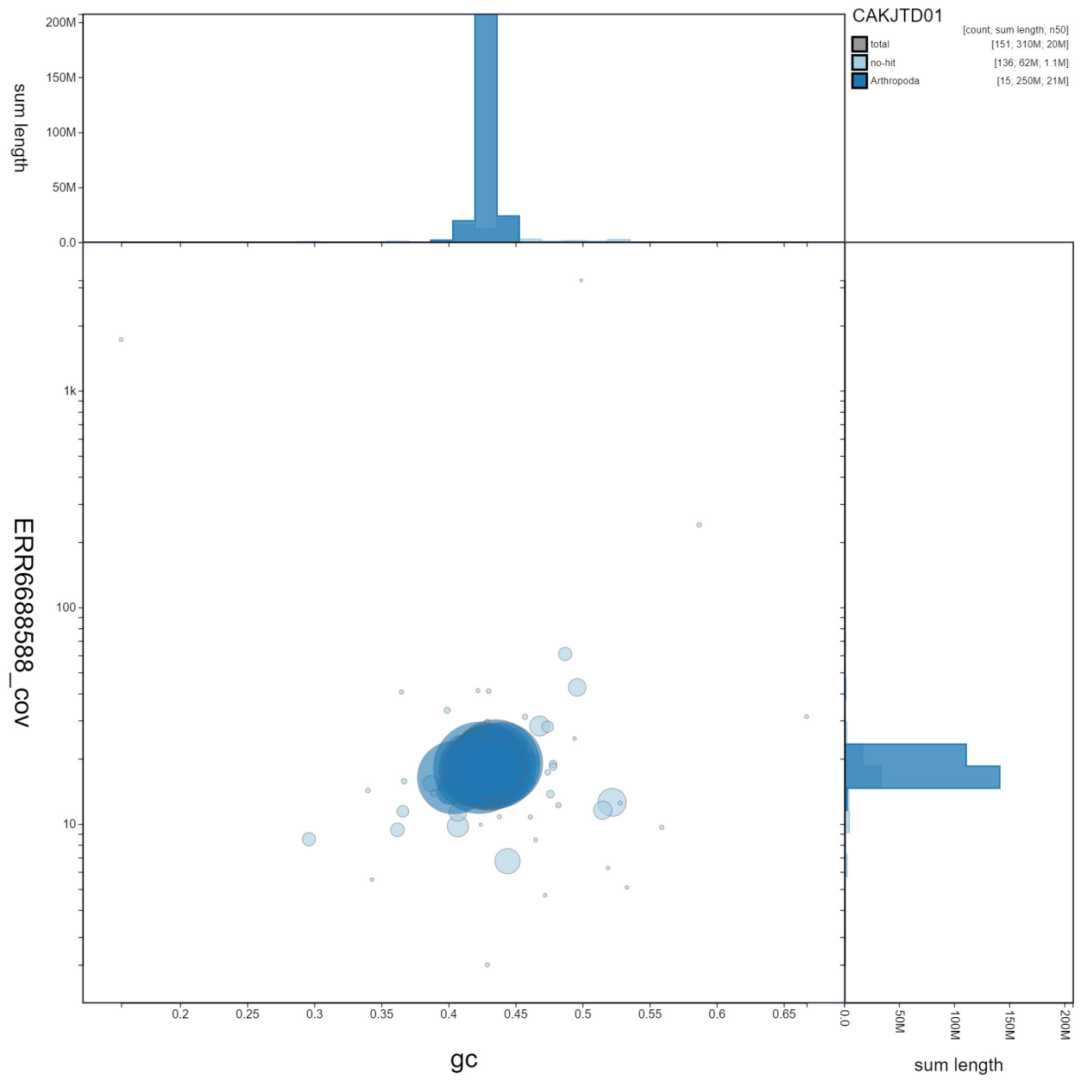
Genome assembly of *Ichneumon xanthorius*, iyIchXant1.1. GC coverage. BlobToolKit GC-coverage plot. Scaffolds are coloured by phylum. Circles are sized in proportion to scaffold length. Histograms show the distribution of scaffold length sum along each axis. An interactive version of this figure is available at https://blobtoolkit.genomehubs.org/view/iyIchXant1.1/dataset/CAKJTD01/blob.

**Figure 4.**
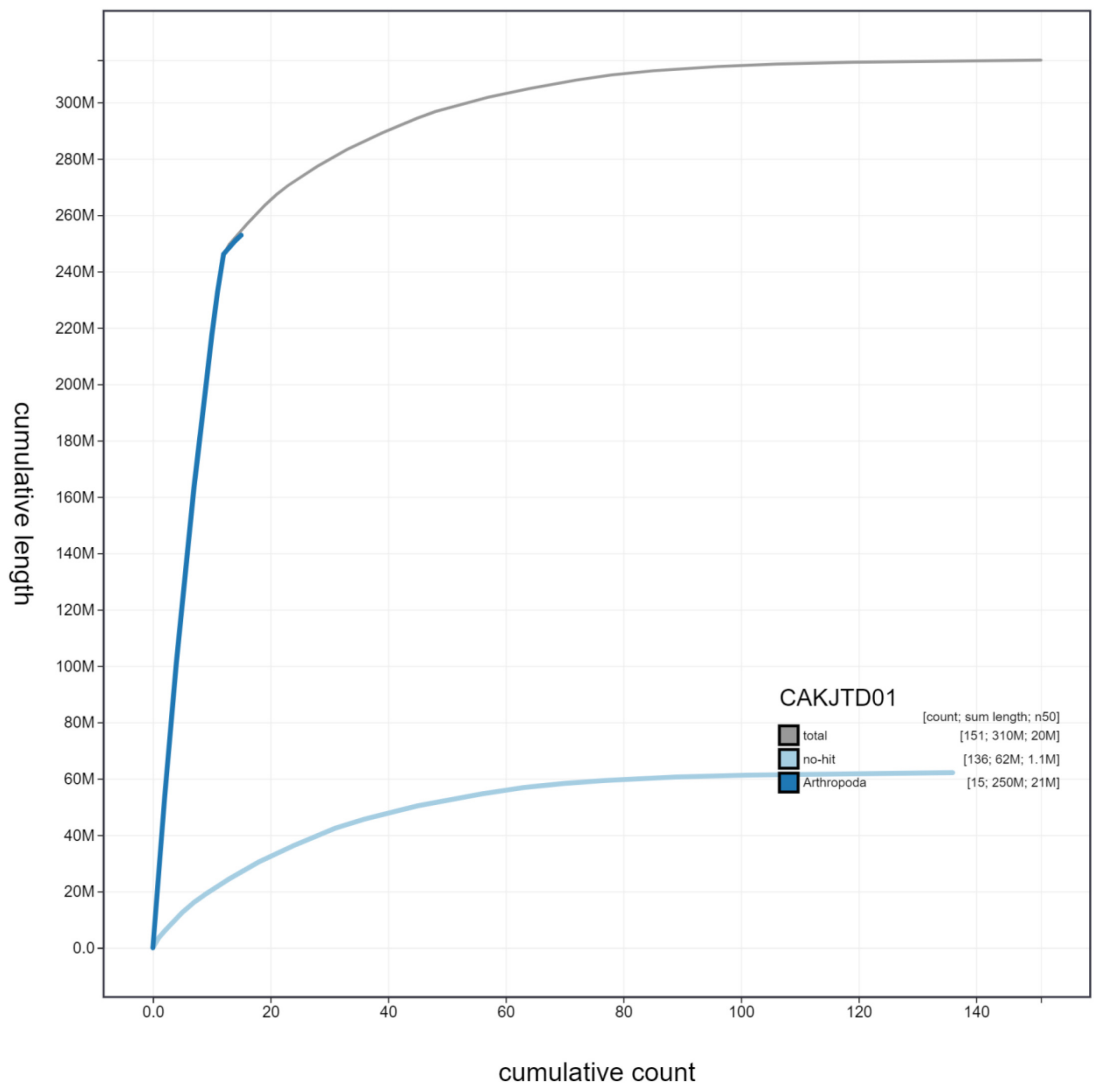
Genome assembly of *Ichneumon xanthorius*, iyIchXant1.1: cumulative sequence. BlobToolKit cumulative sequence plot. The grey line shows cumulative length for all scaffolds. Coloured lines show cumulative lengths of scaffolds assigned to each phylum using the buscogenes taxrule. An interactive version of this figure is available at https://blobtoolkit.genomehubs.org/view/iyIchXant1.1/dataset/CAKJTD01/cumulative.

**Figure 5.**
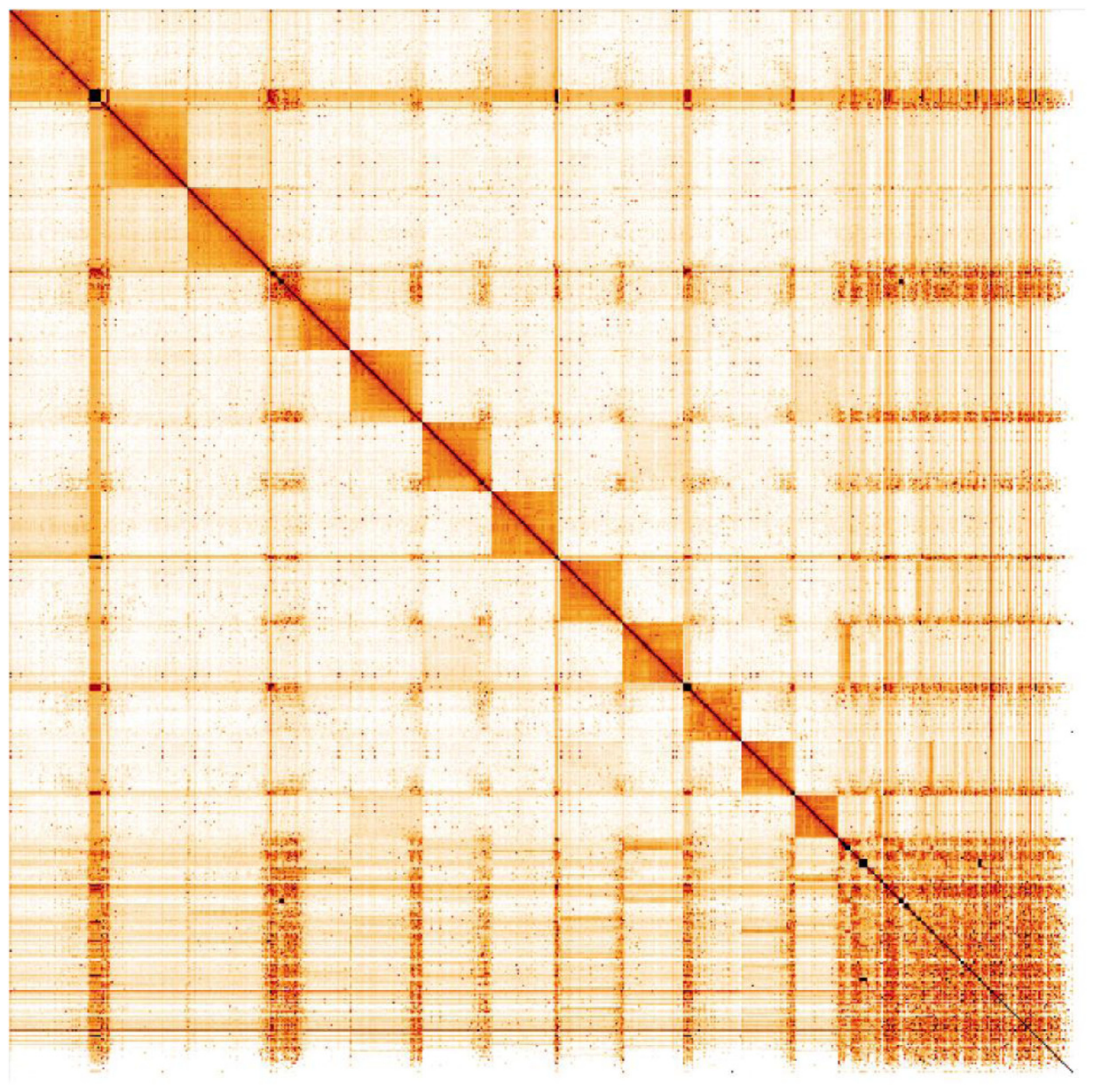
Genome assembly of *Ichneumon xanthorius*, iyIchXant1.1: Hi-C contact map. Hi-C contact map of the iyIchXant1.1 assembly, visualised in HiGlass. Chromosomes are shown in size order from left to right and top to bottom.

**Table 2.** Chromosomal pseudomolecules in the genome assembly of *Ichneumon xanthorius*, iyIchXant1.1.

INSDC accession	Chromosome	Size (Mb)	GC%
OU824200.1	1	27.40	42.3
OU824201.1	2	23.86	43.0
OU824202.1	3	25.76	43.5
OU824203.1	4	24.20	43.3
OU824204.1	5	20.15	42.5
OU824205.1	6	18.22	42.9
OU824206.1	7	21.55	44.0
OU824207.1	8	18.38	42.1
OU824208.1	9	20.52	42.9
OU824209.1	10	17.22	40.4
OU824210.1	11	15.71	42.2
OU824211.1	12	13.19	43.2
OU824212.1	MT	0.02	15.7
-	Unplaced	68.81	43.3

## Genome annotation report

The iyIchXant1.1 genome has been annotated using the Ensembl rapid annotation pipeline ( Table 1; https://rapid.ensembl.org/Ichneumon_xanthorius_GCA_917499995.1/). The resulting annotation includes 17,487 transcribed mRNAs from 10,622 protein-coding and 1,584 non-coding genes. There are 1.49 coding transcripts per gene and 6.14 exons per transcript.

## Methods

### Sample acquisition and DNA extraction

A single female *I. xanthorius* was collected from Wytham Woods, Oxfordshire, UK (latitude 51.774, longitude -1.332) by Liam Crowley, University of Oxford, using a net. The sample was identified by Gavin Broad, Natural History Museum, and snap-frozen on dry ice.

DNA was extracted at the Tree of Life laboratory, Wellcome Sanger Institute. The iyIchXant1 sample was weighed and dissected on dry ice with tissue set aside for Hi-C sequencing. Thorax tissue was disrupted using a Nippi Powermasher fitted with a BioMasher pestle. Fragment size analysis of 0.01–0.5 ng of DNA was then performed using an Agilent FemtoPulse. High molecular weight (HMW) DNA was extracted using the Qiagen MagAttract HMW DNA extraction kit. Low molecular weight DNA was removed from a 200-ng aliquot of extracted DNA using 0.8X AMpure XP purification kit prior to 10X Chromium sequencing; a minimum of 50 ng DNA was submitted for 10X sequencing. HMW DNA was sheared into an average fragment size between 12–20 kb in a Megaruptor 3 system with speed setting 30. Sheared DNA was purified by solid-phase reversible immobilisation using AMPure PB beads with a 1.8X ratio of beads to sample to remove the shorter fragments and concentrate the DNA sample. The concentration of the sheared and purified DNA was assessed using a Nanodrop spectrophotometer and Qubit Fluorometer and Qubit dsDNA High Sensitivity Assay kit. Fragment size distribution was evaluated by running the sample on the FemtoPulse system.

### Sequencing

Pacific Biosciences HiFi circular consensus and 10X Genomics read cloud sequencing libraries were constructed according to the manufacturers’ instructions. Sequencing was performed by the Scientific Operations core at the Wellcome Sanger Institute on Pacific Biosciences SEQUEL II and Illumina NovaSeq 6000 instruments. Hi-C data were generated from head tissue using the Arima v2.0 kit and sequenced on an Illumina NovaSeq 6000 instrument.

### Genome assembly

Assembly was carried out with Hifiasm ( Cheng *et al.*, 2021). Haplotypic duplication was identified and removed with purge_dups ( Guan *et al.*, 2020). Scaffolding with Hi-C data ( Rao *et al.*, 2014) was carried out with SALSA2 ( Ghurye *et al.*, 2019). The Hi-C scaffolded assembly was polished with the 10X Genomics Illumina data by aligning to the assembly with longranger align, calling variants with freebayes ( Garrison & Marth, 2012). One round of the Illumina polishing was applied. The mitochondrial genome was assembled with MitoHiFi ( Uliano-Silva *et al.*, 2021), which performed annotation using MitoFinder ( Allio *et al.*, 2020). The assembly was checked for contamination as described previously ( Howe *et al.*, 2021). Manual curation ( Howe *et al.*, 2021) was performed using HiGlass ( Kerpedjiev *et al.*, 2018) and Pretext. The genome was analysed within the BlobToolKit environment ( Challis *et al.*, 2020). Table 3 contains a list of all software tool versions used, where appropriate.

**Table 3.** Software tools used.

Software tool	Version	Source
Hifiasm	0.15.3	Cheng *et al.*, 2021
purge_dups	1.2.3	Guan *et al.*, 2020
SALSA2	2.2	Ghurye *et al.*, 2019
longranger align	2.2.2	https://support.10xgenomics. com/genome-exome/software/ pipelines/latest/advanced/ other-pipelines
freebayes	v1.3.1-17- gaa2ace8	Garrison & Marth, 2012
MitoHiFi	2	https://github.com/ marcelauliano/MitoHiFi
HiGlass	1.11.6	Kerpedjiev *et al.*, 2018
PretextView	0.0.4	https://github.com/wtsi-hpag/ PretextView
BlobToolKit	2.6.4	Challis *et al.*, 2020

### Genome annotation

The Ensembl gene annotation system ( Aken *et al*., 2016) was used to generate annotation for the Ichneumon xanthorius assembly (GCA_917499995.1). Annotation was created primarily through alignment of transcriptomic data to the genome, with gap filling via protein to-genome alignments of a select set of proteins from UniProt (PMID: 30395287).

### Ethics/compliance issues

The materials that have contributed to this genome note have been supplied by a Darwin Tree of Life Partner. The submission of materials by a Darwin Tree of Life Partner is subject to the Darwin Tree of Life Project Sampling Code of Practice. By agreeing with and signing up to the Sampling Code of Practice, the Darwin Tree of Life Partner agrees they will meet the legal and ethical requirements and standards set out within this document in respect of all samples acquired for, and supplied to, the Darwin Tree of Life Project. Each transfer of samples is further undertaken according to a Research Collaboration Agreement or Material Transfer Agreement entered into by the Darwin Tree of Life Partner, Genome Research Limited (operating as the Wellcome Sanger Institute), and in some circumstances other Darwin Tree of Life collaborators.

## Data availability

European Nucleotide Archive: Ichneumon xanthorius. Accession number PRJEB46331; https://identifiers.org/ena.embl/PRJEB46331.

The genome sequence is released openly for reuse. The *I. xanthorius* genome sequencing initiative is part of the Darwin Tree of Life (DToL) project. All raw sequence data and the assembly have been deposited in INSDC databases. Raw data and assembly accession identifiers are reported in Table 1.

## Author information

Members of the University of Oxford and Wytham Woods Genome Acquisition Lab are listed here: https://doi.org/10.5281/zenodo.5746938.

Members of the Darwin Tree of Life Barcoding collective are listed here: https://doi.org/10.5281/zenodo.5744972.

Members of the Wellcome Sanger Institute Tree of Life programme are listed here: https://doi.org/10.5281/zenodo.5744840.

Members of Wellcome Sanger Institute Scientific Operations: DNA Pipelines collective are listed here: https://doi.org/10.5281/zenodo.5746904.

Members of the Tree of Life Core Informatics collective are listed here: https://doi.org/10.5281/zenodo.5743293.

Members of the Darwin Tree of Life Consortium are listed here: https://doi.org/10.5281/zenodo.5638618.
