# Supplementary material for: The genome sequence of a parasitoid wasp, Ichneumon xanthorius Forster, 1771
Source: Wellcome Open Res. 2022 Feb 10;7:47. [Version 1] doi: 10.12688/wellcomeopenres.17683.1 (PMC8984213; doi:10.12688/wellcomeopenres.17683.1)
Supplement: Peer review history

# Peer review: Reviewer response for version 1

**DOI:** 10.21956/wellcomeopenres.19565.r49264

**Published:** 2022-04-05

**Authors:** Gaelen Burke

**Affiliations:** 1 Department of Entomology, University of Georgia, Athens, GA, USA

This data note describes the genome sequence of *Ichneumon xanthorius* Forster. The genome has been sequenced from a single wasp and is assembled at a chromosomal level. The methodology is described adequately and was based on a PacBio HiFi long reads and scaffolding with a HiC chromatin contacts strategy. Based upon the assembly statistics, the high percentage of complete BUSCO genes and the blobplot that shows very consistent GC and coverage, the genome appears to be very high quality. The genome was annotated with the Ensembl pipeline. The genome size and number of genes, etc., are all similar to other parasitoid genomes currently in the literature. Overall, this genome sequence will be a very useful contribution to the community and to the diversity of genome sequences available from Darwin’s Wasps.

 While another reviewer suggested an analysis to look for evidence of PDVs, I believe that is beyond the scope of this data note. Perhaps the authors could state whether this species was expected to have PDVs *a priori* in the introduction.

Are sufficient details of methods and materials provided to allow replication by others?

Yes

Is the rationale for creating the dataset(s) clearly described?

Yes

Are the datasets clearly presented in a useable and accessible format?

Yes

Are the protocols appropriate and is the work technically sound?

Yes

Reviewer Expertise:

Insect genomics, endogenous viruses

I confirm that I have read this submission and believe that I have an appropriate level of expertise to confirm that it is of an acceptable scientific standard.

# Peer review: Reviewer response for version 1

**DOI:** 10.21956/wellcomeopenres.19565.r49269

**Published:** 2022-04-05

**Authors:** James B. Whitfield

**Affiliations:** 1 Department of Entomology, University of Illinois at Urbana-Champaign, Urbana, IL, USA

This genome report describes the genome sequence of the parasitic wasp, *Ichneumon xanthorius*, and represents the first chromosomal-level genome available for an ichneumonid wasp (the Ichneumonoidae is possibly the largest family of insects). The rationale for why this genome was targeted for sequencing is clearly, although minimally, laid out.

 One briefly mentioned characteristic of the wasp that is of comparative interest is that it is an idiobiont endoparasitoid of host pupae. As an endoparasitoid, it is likely to have some strategy for evading the immune system of its host insect. Most genomic studies of endoparasitoids have been of koinobiont species that attack host larvae, and with associations with symbiotic viruses that are involved with these immunological interactions. It would thus be of interest whether this ichneumonid also possesses a similar virus, or whether it has some other battery of genes for host immune evasion. There are some known (published) core viral gene sequences from the viruses that are housed within other ichneumonid wasps (Campopleginae, Banchinae) that could be used as probes (although it is possible of course that if this wasp species does carry a virus, it might be too distantly related to the other viruses to recognize).

Are sufficient details of methods and materials provided to allow replication by others?

Yes

Is the rationale for creating the dataset(s) clearly described?

Yes

Are the datasets clearly presented in a useable and accessible format?

Yes

Are the protocols appropriate and is the work technically sound?

Yes

Reviewer Expertise:

Taxonomy and systematics of parasitoid wasps, comparative genomics of parasitoid viruses

I confirm that I have read this submission and believe that I have an appropriate level of expertise to confirm that it is of an acceptable scientific standard.

# Peer review: Reviewer response for version 1

**DOI:** 10.21956/wellcomeopenres.19565.r49254

**Published:** 2022-03-17

**Authors:** Xinhai Ye

**Affiliations:** 1 Institute of Insect Sciences, Shanghai Institute for Advanced Study, Zhejiang University, Hangzhou, China

This paper reports a chromosome-level genome of the ichneumonid wasp, *Ichneumon xanthorius. *This genomic resource will greatly facilitates future analyses of the evolution in parasitoid wasps. The sequencing strategies, related bioinformatic methods and genome information are clearly presented and I have only minor comments to the authors:

1. Parasitoid wasps in superfamily Ichneumonoidea often carry polydnaviruses (PDVs) as an important factor in parasitoid-host interaction, and PDV genomes are stably integrated into the genomes of parasitoid wasps. Therefore, I suggest that the authors indicate whether this species ( *Ichneumon xanthorius*) carries PDVs, and this is very useful for the users of this genomic data.
2. Is there any experimental evidence (for example, chromosome staining) to support the 12 chromosomes assembled by SALSA2?

Are sufficient details of methods and materials provided to allow replication by others?

Yes

Is the rationale for creating the dataset(s) clearly described?

Yes

Are the datasets clearly presented in a useable and accessible format?

Yes

Are the protocols appropriate and is the work technically sound?

Yes

Reviewer Expertise:

Genomics, Genome sequencing, Comparative genomics, Insect genomics, Parasitoid wasp genomics, venom

I confirm that I have read this submission and believe that I have an appropriate level of expertise to confirm that it is of an acceptable scientific standard.
